# Review of HIV testing at a district general hospital in an area of high HIV prevalence following the introduction of new national guidelines

**DOI:** 10.1186/1758-2652-13-S4-P218

**Published:** 2010-11-08

**Authors:** ID Page, M Phillips, P Flegg, R Palmer

**Affiliations:** 1 North Manchester General Hospital, Infectious Diseases, Manchester, UK; 2 Trinity College, Genito-Urinary Medicine, Dublin, Ireland; 3 Blackpool Victoria Hospital, Medicine, Blackpool, UK; 4 Blackpool Victoria Hospital, Microbiology, Blackpool, UK

## 

UK national HIV testing guidelines were released in September 2008 and disseminated in our trust through emails to consultants and a grand round presentation. These aimed to promote patient HIV testing by all clinicians regardless of specialty and to trigger testing when specific indicator diseases were diagnosed. The guidelines also state that testing should be considered in all general medical admissions in areas where HIV prevalence exceeds 2 in 1000 of the population. Our audit looked at HIV testing at Blackpool Victoria Hospital (which is located in a region of high HIV prevalence) from October 2007 to September 2009. This represents 1 year before and 1 year after the publication of the new guidelines.

We used our laboratory database to identify proven cases of common diseases where HIV testing is indicated. We then cross referenced this against records of HIV tests performed. We also took the total number of HIV tests requested from the medical wards and compared this to the number of acute medical admissions. We found that in the year after guidelines were published and disseminated within the trust the rate of HIV testing for indicator diseases was as followed:- hepatitis B 6%, hepatitis C 28%, tuberculosis 9%, lymphoma 14%. In the case of hepatitis this represented a decrease on previous years. The overall rate of HIV testing in acute medical admissions was 0.5%.

Our results demonstrate that in our trust traditional methods of guideline dissemination did not lead to effective implementation on this occasion. We are now assessing alternative methods such as marking all positive laboratory results for indicator diseases with the phrase 'HIV TESTING SHOULD BE CONSIDERED' and the possible implementation of universal opt out screening in our Clinical Decisions Unit for all acute general medical admissions.

